# Supplementary material for: Pyruvate Dehydrogenase Kinase 1 inhibition mediated oxidative phosphorylation enhancement in cartilage promotes osteoarthritis progression
Source: BMC Musculoskelet Disord. 2023 Jul 20;24:597. doi: 10.1186/s12891-023-06585-6 (PMC10357736; doi:10.1186/s12891-023-06585-6)
Supplement: Supplementary file 1 — Additional file 1. [file 12891_2023_6585_MOESM1_ESM.zip › KEGG.Gsea.1653622432464/KEGG_LEISHMANIA_INFECTION.html]

Details for gene set KEGG\_LEISHMANIA\_INFECTION[GSEA]

|  || Dataset | 55457\_collapsed\_to\_symbols.cls.cls #OA\_versus\_normal.cls.cls #OA\_versus\_normal\_repos |
| Phenotype | cls.cls#OA\_versus\_normal\_repos |
| Upregulated in class | normal |
| GeneSet | KEGG\_LEISHMANIA\_INFECTION |
| Enrichment Score (ES) | -0.22010814 |
| Normalized Enrichment Score (NES) | -0.8920158 |
| Nominal p-value | 0.6601626 |
| FDR q-value | 0.84119606 |
| FWER p-Value | 1.0 |
Table: GSEA Results Summary

  

Fig 1: Enrichment plot: KEGG\_LEISHMANIA\_INFECTION      
 Profile of the Running ES Score & Positions of GeneSet Members on the Rank Ordered List

  

| SYMBOL | TITLE | RANK IN GENE LIST | RANK METRIC SCORE | RUNNING ES | CORE ENRICHMENT || 1 | CYBA | cytochrome b-245 alpha chain [Source:HGNC Symbol;Acc:HGNC:2577] | 501 | 0.658 | -0.0072 | Yes |
| 2 | FCGR1A | Fc fragment of IgG receptor Ia [Source:HGNC Symbol;Acc:HGNC:3613] | 633 | 0.610 | 0.0121 | Yes |
| 3 | HLA-DMB | "major histocompatibility complex, class II, DM beta [Source:HGNC Symbol;Acc:HGNC:4935]" | 679 | 0.598 | 0.0376 | Yes |
| 4 | MAPK3 | mitogen-activated protein kinase 3 [Source:HGNC Symbol;Acc:HGNC:6877] | 757 | 0.579 | 0.0596 | Yes |
| 5 | ITGB2 | integrin subunit beta 2 [Source:HGNC Symbol;Acc:HGNC:6155] | 827 | 0.562 | 0.0814 | Yes |
| 6 | IFNGR2 | interferon gamma receptor 2 [Source:HGNC Symbol;Acc:HGNC:5440] | 861 | 0.552 | 0.1055 | Yes |
| 7 | TAB2 | TGF-beta activated kinase 1 (MAP3K7) binding protein 2 [Source:HGNC Symbol;Acc:HGNC:17075] | 877 | 0.549 | 0.1309 | Yes |
| 8 | HLA-DQA1 | "major histocompatibility complex, class II, DQ alpha 1 [Source:HGNC Symbol;Acc:HGNC:4942]" | 1334 | 0.452 | 0.1173 | Yes |
| 9 | FCGR2C | Fc fragment of IgG receptor IIc (gene/pseudogene) [Source:HGNC Symbol;Acc:HGNC:15626] | 1460 | 0.435 | 0.1285 | Yes |
| 10 | MAP3K7 | mitogen-activated protein kinase kinase kinase 7 [Source:HGNC Symbol;Acc:HGNC:6859] | 1701 | 0.399 | 0.1291 | Yes |
| 11 | HLA-DMA | "major histocompatibility complex, class II, DM alpha [Source:HGNC Symbol;Acc:HGNC:4934]" | 1769 | 0.388 | 0.1427 | Yes |
| 12 | ITGA4 | integrin subunit alpha 4 [Source:HGNC Symbol;Acc:HGNC:6140] | 1854 | 0.379 | 0.1545 | Yes |
| 13 | TNF | tumor necrosis factor [Source:HGNC Symbol;Acc:HGNC:11892] | 2098 | 0.350 | 0.1525 | Yes |
| 14 | HLA-DOB | "major histocompatibility complex, class II, DO beta [Source:HGNC Symbol;Acc:HGNC:4937]" | 2147 | 0.344 | 0.1654 | Yes |
| 15 | NCF1 | neutrophil cytosolic factor 1 [Source:HGNC Symbol;Acc:HGNC:7660] | 2195 | 0.339 | 0.1781 | Yes |
| 16 | MAPK14 | mitogen-activated protein kinase 14 [Source:HGNC Symbol;Acc:HGNC:6876] | 2352 | 0.320 | 0.1815 | Yes |
| 17 | TGFB1 | transforming growth factor beta 1 [Source:HGNC Symbol;Acc:HGNC:11766] | 2490 | 0.303 | 0.1855 | Yes |
| 18 | MAPK13 | mitogen-activated protein kinase 13 [Source:HGNC Symbol;Acc:HGNC:6875] | 2544 | 0.298 | 0.1957 | Yes |
| 19 | C3 | complement C3 [Source:HGNC Symbol;Acc:HGNC:1318] | 2764 | 0.273 | 0.1919 | Yes |
| 20 | HLA-DQB1 | "major histocompatibility complex, class II, DQ beta 1 [Source:HGNC Symbol;Acc:HGNC:4944]" | 2840 | 0.266 | 0.1989 | Yes |
| 21 | NCF4 | neutrophil cytosolic factor 4 [Source:HGNC Symbol;Acc:HGNC:7662] | 3036 | 0.246 | 0.1956 | No |
| 22 | ELK1 | ETS transcription factor ELK1 [Source:HGNC Symbol;Acc:HGNC:3321] | 3357 | 0.216 | 0.1812 | No |
| 23 | PTPN6 | protein tyrosine phosphatase non-receptor type 6 [Source:HGNC Symbol;Acc:HGNC:9658] | 3434 | 0.208 | 0.1853 | No |
| 24 | RELA | "RELA proto-oncogene, NF-kB subunit [Source:HGNC Symbol;Acc:HGNC:9955]" | 3942 | 0.161 | 0.1536 | No |
| 25 | JAK2 | Janus kinase 2 [Source:HGNC Symbol;Acc:HGNC:6192] | 4183 | 0.140 | 0.1416 | No |
| 26 | HLA-DOA | "major histocompatibility complex, class II, DO alpha [Source:HGNC Symbol;Acc:HGNC:4936]" | 4344 | 0.125 | 0.1352 | No |
| 27 | STAT1 | signal transducer and activator of transcription 1 [Source:HGNC Symbol;Acc:HGNC:11362] | 4495 | 0.113 | 0.1290 | No |
| 28 | FCGR3A | Fc fragment of IgG receptor IIIa [Source:HGNC Symbol;Acc:HGNC:3619] | 4594 | 0.105 | 0.1264 | No |
| 29 | NFKB1 | nuclear factor kappa B subunit 1 [Source:HGNC Symbol;Acc:HGNC:7794] | 4626 | 0.102 | 0.1289 | No |
| 30 | HLA-DPB1 | "major histocompatibility complex, class II, DP beta 1 [Source:HGNC Symbol;Acc:HGNC:4940]" | 5486 | 0.033 | 0.0636 | No |
| 31 | TAB1 | TGF-beta activated kinase 1 (MAP3K7) binding protein 1 [Source:HGNC Symbol;Acc:HGNC:18157] | 5684 | 0.017 | 0.0490 | No |
| 32 | TRAF6 | TNF receptor associated factor 6 [Source:HGNC Symbol;Acc:HGNC:12036] | 5761 | 0.011 | 0.0437 | No |
| 33 | ITGAM | integrin subunit alpha M [Source:HGNC Symbol;Acc:HGNC:6149] | 5820 | 0.007 | 0.0395 | No |
| 34 | IL12B | interleukin 12B [Source:HGNC Symbol;Acc:HGNC:5970] | 5991 | -0.004 | 0.0264 | No |
| 35 | TGFB3 | transforming growth factor beta 3 [Source:HGNC Symbol;Acc:HGNC:11769] | 6293 | -0.027 | 0.0043 | No |
| 36 | FCGR2A | Fc fragment of IgG receptor IIa [Source:HGNC Symbol;Acc:HGNC:3616] | 6329 | -0.031 | 0.0030 | No |
| 37 | IFNG | interferon gamma [Source:HGNC Symbol;Acc:HGNC:5438] | 6410 | -0.036 | -0.0015 | No |
| 38 | PRKCB | protein kinase C beta [Source:HGNC Symbol;Acc:HGNC:9395] | 6453 | -0.039 | -0.0029 | No |
| 39 | HLA-DRA | "major histocompatibility complex, class II, DR alpha [Source:HGNC Symbol;Acc:HGNC:4947]" | 6550 | -0.046 | -0.0082 | No |
| 40 | MYD88 | MYD88 innate immune signal transduction adaptor [Source:HGNC Symbol;Acc:HGNC:7562] | 6638 | -0.052 | -0.0124 | No |
| 41 | IL4 | interleukin 4 [Source:HGNC Symbol;Acc:HGNC:6014] | 6673 | -0.054 | -0.0124 | No |
| 42 | MAPK1 | mitogen-activated protein kinase 1 [Source:HGNC Symbol;Acc:HGNC:6871] | 6853 | -0.068 | -0.0231 | No |
| 43 | NCF2 | neutrophil cytosolic factor 2 [Source:HGNC Symbol;Acc:HGNC:7661] | 6871 | -0.069 | -0.0211 | No |
| 44 | IL12A | interleukin 12A [Source:HGNC Symbol;Acc:HGNC:5969] | 6930 | -0.074 | -0.0220 | No |
| 45 | TGFB2 | transforming growth factor beta 2 [Source:HGNC Symbol;Acc:HGNC:11768] | 6985 | -0.079 | -0.0224 | No |
| 46 | JAK1 | Janus kinase 1 [Source:HGNC Symbol;Acc:HGNC:6190] | 6995 | -0.079 | -0.0193 | No |
| 47 | HLA-DRB1 | "major histocompatibility complex, class II, DR beta 1 [Source:HGNC Symbol;Acc:HGNC:4948]" | 7087 | -0.086 | -0.0222 | No |
| 48 | IFNGR1 | interferon gamma receptor 1 [Source:HGNC Symbol;Acc:HGNC:5439] | 7176 | -0.094 | -0.0245 | No |
| 49 | MARCKSL1 | MARCKS like 1 [Source:HGNC Symbol;Acc:HGNC:7142] | 7621 | -0.129 | -0.0529 | No |
| 50 | TLR4 | toll like receptor 4 [Source:HGNC Symbol;Acc:HGNC:11850] | 7703 | -0.134 | -0.0527 | No |
| 51 | IL1A | interleukin 1 alpha [Source:HGNC Symbol;Acc:HGNC:5991] | 7842 | -0.144 | -0.0565 | No |
| 52 | TLR2 | toll like receptor 2 [Source:HGNC Symbol;Acc:HGNC:11848] | 7991 | -0.154 | -0.0606 | No |
| 53 | MAPK12 | mitogen-activated protein kinase 12 [Source:HGNC Symbol;Acc:HGNC:6874] | 8161 | -0.168 | -0.0656 | No |
| 54 | IL10 | interleukin 10 [Source:HGNC Symbol;Acc:HGNC:5962] | 8855 | -0.221 | -0.1089 | No |
| 55 | FCGR3B | Fc fragment of IgG receptor IIIb [Source:HGNC Symbol;Acc:HGNC:3620] | 9341 | -0.259 | -0.1342 | No |
| 56 | IRAK1 | interleukin 1 receptor associated kinase 1 [Source:HGNC Symbol;Acc:HGNC:6112] | 9377 | -0.262 | -0.1243 | No |
| 57 | HLA-DPA1 | "major histocompatibility complex, class II, DP alpha 1 [Source:HGNC Symbol;Acc:HGNC:4938]" | 9481 | -0.273 | -0.1191 | No |
| 58 | FOS | "Fos proto-oncogene, AP-1 transcription factor subunit [Source:HGNC Symbol;Acc:HGNC:3796]" | 10640 | -0.387 | -0.1906 | No |
| 59 | IRAK4 | interleukin 1 receptor associated kinase 4 [Source:HGNC Symbol;Acc:HGNC:17967] | 10874 | -0.413 | -0.1888 | No |
| 60 | NFKBIB | NFKB inhibitor beta [Source:HGNC Symbol;Acc:HGNC:7798] | 11277 | -0.468 | -0.1975 | No |
| 61 | CR1 | complement C3b/C4b receptor 1 (Knops blood group) [Source:HGNC Symbol;Acc:HGNC:2334] | 11376 | -0.483 | -0.1818 | No |
| 62 | MAPK11 | mitogen-activated protein kinase 11 [Source:HGNC Symbol;Acc:HGNC:6873] | 11501 | -0.501 | -0.1672 | No |
| 63 | IL1B | interleukin 1 beta [Source:HGNC Symbol;Acc:HGNC:5992] | 12105 | -0.622 | -0.1841 | No |
| 64 | NOS2 | nitric oxide synthase 2 [Source:HGNC Symbol;Acc:HGNC:7873] | 12262 | -0.665 | -0.1641 | No |
| 65 | ITGB1 | integrin subunit beta 1 [Source:HGNC Symbol;Acc:HGNC:6153] | 12566 | -0.780 | -0.1499 | No |
| 66 | HLA-DRB4 | "major histocompatibility complex, class II, DR beta 4 [Source:HGNC Symbol;Acc:HGNC:4952]" | 12636 | -0.815 | -0.1159 | No |
| 67 | PTGS2 | prostaglandin-endoperoxide synthase 2 [Source:HGNC Symbol;Acc:HGNC:9605] | 12641 | -0.820 | -0.0765 | No |
| 68 | JUN | "Jun proto-oncogene, AP-1 transcription factor subunit [Source:HGNC Symbol;Acc:HGNC:6204]" | 12813 | -0.994 | -0.0418 | No |
| 69 | NFKBIA | NFKB inhibitor alpha [Source:HGNC Symbol;Acc:HGNC:7797] | 12817 | -1.001 | 0.0065 | No |
Table: GSEA details [plain text format]

  

Fig 2: KEGG\_LEISHMANIA\_INFECTION      
 Blue-Pink O' Gram in the Space of the Analyzed GeneSet

  

Fig 3: KEGG\_LEISHMANIA\_INFECTION: Random ES distribution      
 Gene set null distribution of ES for **KEGG\_LEISHMANIA\_INFECTION**
